# The Role of Upregulated APOE in Alzheimer’s Disease Etiology

**DOI:** 10.4172/2161-0460.1000209

**Published:** 2016-02-09

**Authors:** William K Gottschalk, Mirta Mihovilovic, Allen D Roses, Ornit Chiba-Falek

**Affiliations:** 1 Department of Neurology, Duke University Medical Center, Durham, NC 27710, USA; 2 Center for Genomic and Computational Biology, Duke University Medical Center, Durham, NC 27710, USA; 3 Zinfandel Pharmaceuticals, Chapel Hill, NC, USA

The first and most firmly established genetic risk factor for sporadic late onset Alzheimer’s disease (LOAD) is the e4 allele of the apolipoprotein E (*APOE)* gene [1]. Carrying the *APOE*e4 variant significantly increases the lifetime risk for LOAD, with the number of copies present indicative of level of risk [1,2] and is associated with lower age of clinical disease onset [1,3–6]. Furthermore, genome-wide association studies (GWAS) for sporadic LOAD confirmed that *APOE* is the major susceptibility genomic region for the disease and reported significant associations with markers within the *APOE* linkage disequilibrium (LD) locus (contains *APOE*, *TOMM40* and *APOC1* genes). The strongest association signal (by wide margin) in these studies was found at the *APOE* LD region and no other LOAD-association in the human genome remotely approached the same level of significance [7–10]. However, the molecular mechanism underlying the reported genetic LOAD-associations with *APOE* LD region in general and *APOE*e4 haplotype in particular has yet to be discovered.

It has been suggested that alteration of the expression levels of specific genes may be an important mechanism in the etiology of neurodegenerative disorders including LOAD [11]. Previously, using temporal and occipital tissues obtained from *APOE*e3/3 donors we showed that *APOE*-mRNA levels are significantly increased in LOAD-affected brains compared to controls [12]. In preliminary studies, we performed expression analysis in cortical neurons from the temporal cortex of 3 LOAD patients and 3 normal controls isolated by laser capture microdissection (LCM) technique. We analyzed the *APOE*-mRNA counts relative to geometric mean of two housekeeping genes using the nCounter single cell gene expression technology and the nSolver program (NanoString). The results showed increased *APOE*-mRNA in LOAD compared to normal (our unpublished data) and validated our published findings obtained using homogenates of brain tissue for the expression analysis [12]. Our observation was consistent with other reports of elevated levels of *APOE*-mRNA in LOAD brains. For example, Zarow et al. report increased *APOE*-mRNA levels in the hippocampus of AD cases compared to controls [13] and Matsui et al. report increased *APOE*-mRNA levels in temporal cortex of AD donors compared to controls [14]. Furthermore, Akram et al. have demonstrated that *APOE*-mRNA and protein levels in the inferior temporal gyrus and the hippocampus are strongly, positively correlated with the progression of cognitive dysfunction [15].

A recent study showed that endoplasmic reticulum (ER)-mitochondrial communication and mitochondria associated ER membranes (MAM) function-as measured by the synthesis of phospholipids and of cholesteryl esters, respectively-are increased significantly in cells treated with *APOE*e4-containing astrocyte-conditioned media (ACM) as compared to those treated with *APOE*e3-containing ACM [16]. Upregulated MAM function was implicated in the pathogenesis of AD [17,18]. The new findings that *APOE*e4 protein upregulates the activity of MAM may explain, in part, the contribution of *APOE*e4 as a risk factor in the disease. Enhanced activity of *APOE*e4 protein in correlation to AD-related cellular phenotypes has also been described previously. In human AD brain samples, amyloid deposits correlate with gene dosage of *APOE*e4 [19], and *APOE*e4 protein more actively forms fibrils with Aβ protein than *APOE*e3 *in vitro* [20]; moreover, *APOE*e4 aggregates are themselves neurotoxic [21]. *APOE*e4 is susceptible to cleavage of the C-terminus by cellular proteases, and the C-terminal fragments are cytotoxic, in part by eliciting intracellular neurofibrillary tangle formation and in part via disruption of mitochondrial and cytoskeletal functions [22–24]. *APOE*e4 and *APOE*e3 have different lipid-binding characteristics [25], contributing to greater Aβ-elicited lysosomal leakage and apoptosis in *APOE*e4-producing cells [26], and affecting the respective abilities of *APOE*e3 and *APOE*e4 to support neuronal maintenance and repair.

Interestingly, we showed that SNP rs429358, that defines the *APOE*e4 haplotype, has a significant effect on *APOE*-mRNAs levels in temporal cortex obtain from LOAD cases. We demonstrated that the level of *APOE* mRNA was significantly higher in the *APOE*e3/3 genotype group compared to *APOE*e3/4-genotype (Figure 1). In unpublished work, we measured *APOE*-mRNA levels in whole brains from humanized–*APOE*e3 and –*APOE*e4 homozygous mouse models generated by targeted replacement [27,28]. We found that human *APOE*-mRNA levels are>35% higher in brains of *APOE*e3 homozygous mice compared to mice homozygotes to *APOE*e4 (Figure 2). The analysis of humanized-*APOE* mice support the findings in LOAD-human brains, suggesting that while the effect of e4 variant is putatively on increased *activity* of the *APOE* protein, the effect of the e3 background is possibly executed via regulation of *APOE* gene expression that determines the steady state *amount* of the protein.

Different factors may regulate *APOE* gene expression including, but not limited to, genetic [12,29–31] and epigenetic [32] mechanisms. *Cis*-genetic variably on the background of the e3 haplotype contributes to differential *APOE* gene expression. We reported data showing that 523-polyT genotype, located upstream of *APOE* within the adjutant *TOMM40* locus, affects expression of genes in *APOE* LD region [12]. We demonstrated that the LOAD risk allele, very long (‘VL’), is associated with increased levels of *APOE* transcripts in normal and LOAD-affected human brain tissues and with higher luciferase expression in a cell-based reporter system, compared to the short (‘S’) allele [12]. These observations provide a possible explanation for the genetic association of the 523-polyT locus with age of LOAD onset [33,34] and other disease related phenotypes [35–38]. Our observations were recently reproduced by Payton, et al. They showed that the shorter length poly-T variants act as a repressor of luciferase gene expression in reporter gene constructs, whereas expression was reduced to approximately half of that observed for the ‘VL’ variant [39].

Collectively the studies reviewed here suggest that up-regulated function of *APOE* due to either enhanced protein activity or increased *APOE* expression levels may contribute, in part, to the etiology of LOAD. Figure 3 summarizes our proposed model. While this model suggests the triggering event, the biochemical and cell biological pathways that mediate the consequences of this event are still being determined. Our perception of increased *APOE*e3 protein levels as a LOAD-pathogenic mechanism agrees with the concept that changes in expression levels of ‘normal’ protein in the brain can lead to neurodegenerative diseases. In conclusion, genetic heterogeneity across the *APOE*-LD region may lead, through different molecular mechanisms, to elevated (‘pathogenic’) *ApoE* function and possibly explains the extremely strong genetic association of the *APOE*-LD region with increased LOAD-risk and related phenotypes.

## Figures and Tables

**Figure 1:**
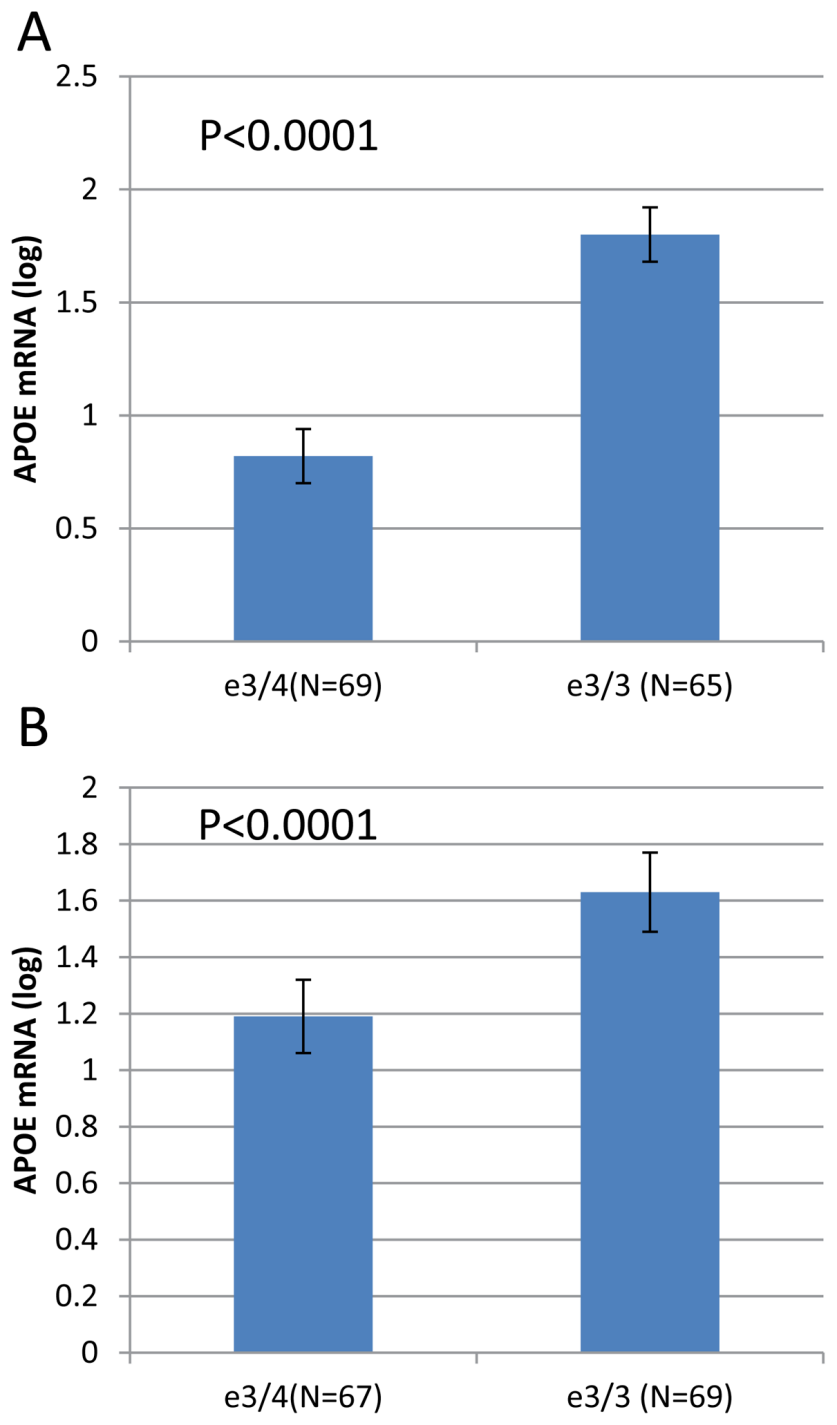
The effect of *APOE* haplotypes on *APOE*-mRNAs expression levels in human brain tissues from LOAD donors The study cohort consisted of brain (temporal and occipital cortex) tissues from Caucasian donors with LOAD. Subjects were genotyped for rs429358 and rs7412 SNPs to determine *APOE* status. Fold levels of human *APOE* mRNA were assayed in (**A**) temporal and (**B**) occipital tissues by real-time RT-PCR using TaqMan technology and calculated relative the geometric mean of *GAPDH-* and *PPIA-*mRNAs reference control using the 2^−ΔΔCt^ method. The expression levels between e3/4 (rs429358-TC) and e3/3 (rs429358-TT) were compared. The values presented here are means levels *±* SE adjusted for age, sex, PMI, and Braak and Braak stage.

**Figure 2:**
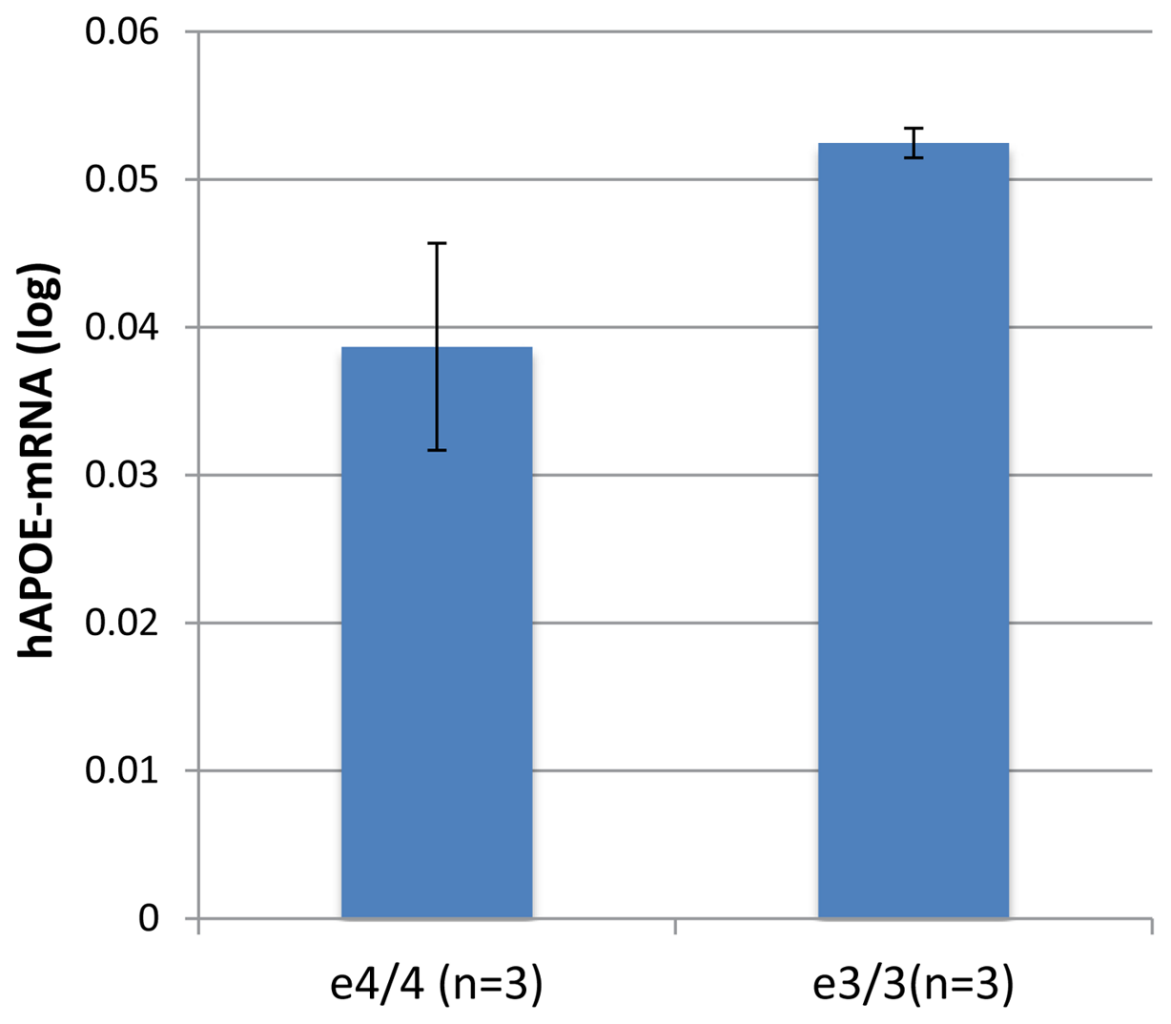
The effect of *APOE* haplotypes on human-*APOE* mRNAs expression levels in humanized mice brain tissues RNA was extracted from whole brain of three mice homozygotes for the human *APOE*e3 and three mice *APOE*e4 homozygous generated by targeted replacement^28^. Fold levels of human *APOE* mRNA were assayed in whole brain tissues by real-time RT-PCR using TaqMan technology and calculated relative the geometric mean of the mouse housekeeping genes, *Gapdh-* and *Ppia-*mRNAs reference control using the 2^−ΔΔCt^ method. The expression levels between e4/4 and e3/3 were compared and the values presented here are means levels *±* SE.

**Figure 3:**
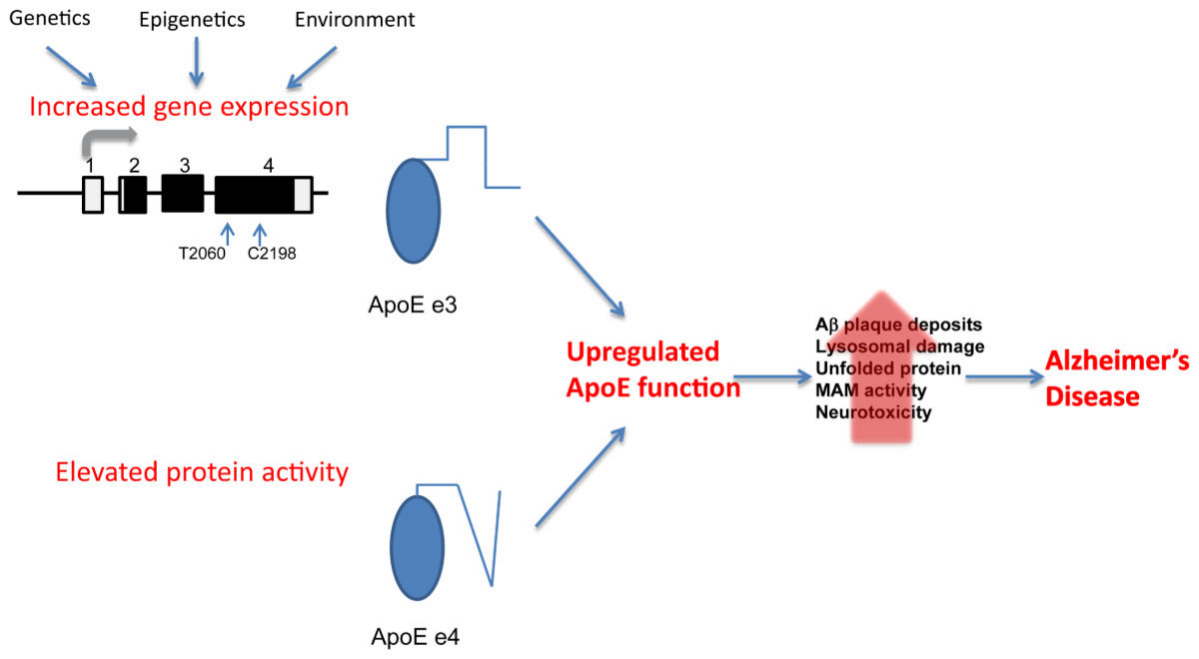
A schematic model describing factors leading to upregulation of ApoE function and the impact on LOAD pathogenesis.
